# The impact of acute low salinity stress on Antarctic echinoderms

**DOI:** 10.1098/rspb.2024.1038

**Published:** 2024-09-18

**Authors:** Nicholas J. Barrett, Elizabeth M. Harper, Lloyd S. Peck

**Affiliations:** ^1^ British Antarctic Survey, Natural Environment Research Council, Cambridge CB3 0ET, UK; ^2^ Department of Earth Sciences, University of Cambridge, Cambridge CB2 3EQ, UK

**Keywords:** osmotic stress, climate change, sea star, sea urchin, sea cucumber, brittle star

## Abstract

Climate change is causing increased coastal freshening in Antarctica, leading to reduced salinity. For Antarctica’s endemic echinoderms, adapted to the stable polar environment, the impact of rapid reductions in coastal salinity on physiology and behaviour is currently unknown. Six common Antarctic echinoderms (the sea urchin *Sterechinus neumayeri*; the sea star *Odontaster validus*; the brittle star *Ophionotus victoriae*; and three sea cucumbers *Cucumaria georgiana*, *Echinopsolus charcoti* and *Heterocucumis steineni*), were directly transferred from ambient salinity (34.5‰) to a range of salinity dilutions (29–9‰) for 24 h. All species showed reduced activity and the establishment of a temporary osmotic gradient between coelomic fluid and external seawater. Most species exhibited a depression in oxygen consumption across tolerated salinities; however, at very low salinities that later resulted in mortality, oxygen consumption increased to levels comparable to those at ambient. Low salinity tolerance varied substantially between species, with *O. victoriae* being the least tolerant (24 h LC_50_ (lethal for 50% of animals) = 19.9‰) while *E. charcoti* and *C. georgiana* demonstrated the greatest tolerance (24 h LC_50_ = 11.5‰). These findings demonstrate the species-specific response of Antarctica’s endemic echinoderms to short-term hypoosmotic salinity events, providing valuable insight into this phylum’s ability to respond to an underreported impact of climate change.

## 1. Introduction

Globally, ecosystems are expected to face widespread impacts due to climate change. In the oceans, the effects of warming and ocean acidification on biodiversity have received considerable attention [1–3]. However, the impact of freshening (the lowering of seawater salinity) leading to osmotic stress in marine organisms has received less focus. Dramatic freshening events are increasing globally as a result of climate change [4], and in polar regions, where there is significant freshwater locked up as ice, warming has the potential to release large amounts into nearshore ecosystems. The Antarctic icesheet, which holds ~70% of global freshwater, is projected to lose mass at an increasing rate throughout the twenty-first century owing to climate change [5]. Warming in West Antarctica and the Western Antarctic Peninsula has already led to increased freshwater influx into the Southern Ocean, resulting in coastal freshening [6–8]. The coastline here has extensive fjordic systems that are currently rich in biodiversity and benthic abundance [9]. However, there is concern that as marine-terminating glaciers retreat owing to warming, the formation of distinct salinity stratification in the water column [10] may threaten these biodiversity hotspots [9]. Antarctic fjords along the Western Antarctic Peninsula are considered to be at an earlier stage of climate warming than Arctic fjords [9]. In Greenland, inner fjord salinities can regularly persist below 25‰ down to approximately 10 m in the summer [11], potentially forecasting similar conditions for Antarctic fjords as climate warming progresses. More acutely, the frequency of intense hyposalinity events—when large volumes of freshwater rapidly enter the marine environment—is increasing owing to climate change [4,5]. For marine animals, salinity is a major environmental variable that impacts their development and survival [12]. Reductions in seawater salinity as a result of freshening can exert stress on marine animals by increasing the cost of osmoregulation [13] and also cause direct cellular damage [14].

Echinoderms are a marine phylum and are generally considered stenohaline with limited control over their extracellular body fluid in response to changes in environmental salinity [15–17]. This is thought to be due to the high permeability of their body wall epithelia and the absence of a specific excretory organ [15,18,19]. However, many echinoderm species live in intertidal habitats where salinity is highly variable, while numerous species are established in brackish and estuarine environments [16,17,20]. Experimental studies have shown that many echinoderms across all classes (with only one example from crinoids) can tolerate short-term, acute reductions in salinity, with some displaying remarkable tolerance [16,17]. For example, Barker & Russell [21] demonstrated that the sea star *Patiriella regularis* was able to survive for 4 days at 0‰. This approach by Barker & Russell [21] and others examines the hypoosmotic shock response to lower salinity for periods typically ranging from 12 to 96 h. This method replicates the rapid salinity changes that animals may experience in environments such as fjord haloclines or during sudden precipitation changes in intertidal zones [21–23]. In polar regions, these events can be extreme, with meltwater from glaciers and ice shelves rapidly introducing substantial amounts of freshwater into intertidal zones, which can persist for extended periods [24]. Here, salinity levels can drop suddenly, occasionally exposing slow-moving benthic invertebrates to pure freshwater [24,25]. Responses to hypoosmotic shock differ from the gradual cellular adjustments that occur as part of the acclimation process when exposed to low salinity over longer periods, such as weeks to months [26–28]. Numerous studies have demonstrated successful acclimation to reduced salinities over longer durations in echinoderms; however, Antarctic species are yet to be examined [20,27,29]. Mechanisms that enable short-term tolerance to reduced salinity in echinoderms may be facilitated through the establishment of transient ionic and osmotic gradients between the extracellular coelomic fluid and the external seawater. These can persist for several hours (between 12 and 24 h [30]), offering protection for internal tissue, which would otherwise rapidly swell on encountering reduced-salinity seawater [27,31,32]. Additionally, some echinoderms appear capable of moderate cell volume or tissue water regulation after hypoosmotic shock, minimizing swelling that would occur without regulation [32].

Many marine animals are endemic to Antarctica, having adapted to the cold, but stable and predictable environmental conditions of the Southern Ocean [33]. Changes in glacial cycles have facilitated periods of high species dispersal around Antarctica, followed by prolonged periods of species isolation in refugia during peak glacial periods. This phenomenon has resulted in a high diversity of colonizing founder species, which have later undergone intraregional speciation aided by the isolation [34]. This, in addition to a lack of shell-crushing predators [35], is thought to have contributed to Antarctica becoming exceptionally rich in echinoderm diversity [34], where they represent around 10% of the benthic fauna [36]. However, the prolonged stability in the Antarctic marine environment may have contributed to a reduced ability for marine species to tolerate rapid change. Their capacity to acclimate to warming temperatures is already known to be poor, while those that can acclimate take substantially longer to do so compared with temperate or tropical species [37]. The ability to tolerate reduced salinities across Antarctic species is less well known. Studies on fish, such as *Harpagifer antarcticus*, demonstrate tolerance to modest drops in salinity, but lower salinities result in osmotic imbalances and an inability to maintain homeostasis [38], dramatically amplified when combined with increased temperature [39]. The limpet *Nacella concinna* demonstrates limited acute tolerance to low salinity [40] and stenohaline characteristics in regards to acclimation to low salinities [41]. Very small reductions in salinity (34–30‰) impair behaviour functions critical to long-term survival in the isopod *Serolis polita* [42]. Multiple studies have demonstrated that low salinity can be a significant modifier of fitness when combined with increased temperature or reduced pH [41,43,44]. For Antarctic echinoderms, there is very little knowledge of their ability to tolerate reduced salinities, with only two studies to date. Pearse [45] demonstrated that the common Antarctic asteroid *Odontaster validus,* was able to maintain body volume on immersion in 50% seawater, suggesting a degree of control over coelomic fluids that would be beneficial on exposure to summer meltwater. Cowart *et al*. [46] demonstrated that embryos of the Antarctic echinoid *Sterechinus neumayeri* were highly sensitive to small reductions in salinity (a decrease from 34 to 30‰ resulted in 99% mortality), suggesting early life stages of Antarctic echinoderms may be extremely vulnerable to coastal freshening. As the majority of Antarctic echinoderms are considered subtidal, and ice restricts the colonization of shallow sites [33], it may be expected that either they will not experience such dramatic shifts in salinity levels, or they will migrate to deeper, higher-salinity waters (e.g. [44]). However, changes in community structure as a result of species migrations away from low-salinity environments may have cascading effects on the entire Antarctic food web [47].

Considering the rapid climate changes underway in Antarctica, it is crucial to evaluate the vulnerability of echinoderms to the impact of freshening events and reduced salinity. This assessment is especially pertinent as species such as *S. neumayeri* and *O. validus* are considered keystone species in Antarctic food webs [47,48], and Antarctic holothurians account for *ca* 10% of global holothurian diversity [34]. In this study, six echinoderms common to the shallow coastal shelf, the echinoid *S. neumayeri*, the asteroid *O. validus*, the ophiuroid *Ophionotus victoriae* and three holothurians, *Heterocucumis steineni, Cucumaria georgiana* and *Echinopsolus charcoti*, were collected from Ryder Bay, on the Antarctic Peninsula. Subtidal salinity here is relatively stable [33.4 ± 0.4‰ at 15 m (mean ± s.d.)], although surface salinity occasionally drops to ~28.5‰ [49]. However, two of these species (*S. neumayeri* and *O. validus*) are occasionally found in the low intertidal (although more common from ~6 m depth [50–52]), where salinities can drop to <12‰ [24,25,38,52]. In this regard, it is hypothesized that these two species would have greater acute hyposalinity tolerance than the four species found only subtidally. All animals were assessed for short-term tolerance to reduced salinities over a 24 h period. Metrics including mortality, oxygen consumption, activity and osmolality of coelomic fluid were measured to evaluate their response.

## 2. Methods

### (a) Experimental animals

All animals were collected in the austral summers of 2020/2021 and 2021/2022 by SCUBA divers at depths of 10–20 m near the British Antarctic Survey’s (BAS) Rothera research station, Adelaide Island (67^o^34’07’’S, 68^o^07^’^30’’W). Salinity and temperature (mean ± s.e.m.) at 15 m depth near the collection site were 33.56 ± 0.06‰ (33.27−33.81‰; *n* = 13) and −0.51 ± 0.16°C (−1.28 to 0.59°C; *n* = 13) from November 2020 to February 2021, and 33.52 ± 0.05‰ (33.01−33.83‰; *n* = 20) and −0.51 ± 0.17°C (−1.58 to 1.24°C; *n* = 20) from November 2021 to February 2022 [49]. After collection, they were transferred to the Rothera aquarium, then transported to the UK via a temperature-controlled containerized aquarium. Once in the UK, they were transferred to the polar aquarium at British Antarctic Survey (BAS) Cambridge and held in 280 l tanks in a recirculating aquarium incorporating macro- and biological filtration systems, using seawater from the UK coast. The aquarium was maintained at −0.3 ± 0.2°C and 34.5‰, with a 12 h light/dark photoperiod for 6–12 months prior to experiments. *Sterechinus neumayeri*, *O. validus* and *O. victoriae* were fed weekly on a frozen krill diet, while the three holothurians were fed weekly on a ground flake diet (Vitalis, Mixed Reef Food). Prior to all experiments, food was restricted for at least 48 h. All animals were maintained and handled per UK animal welfare regulations.

### (b) Experimental set-up

Six salinity treatments were used to assess echinoderm salinity tolerance to an acute stress over a 24 h period: 34.5 (control), 29, 24, 19, 14 and 9‰. Animals were subjected to incrementally lower salinity treatments until the lower lethal concentration for 50% (LC_50_) of the population was reached; 9‰ was not assessed in *S. neumayeri* and *O. victoriae*. Numbers of animals used for each experimental salinity were, 10 each for *S. neumayeri* (total *n* = 50; test diameter 20−34 mm), *O. validus* (total *n* = 60; arm radius from the tip of arm to the centre of mouth 36−44 mm) and *O. victoriae* (*n* = 50; central disc diameter 16−31 mm), and eight for *H. steineni* (total *n* = 48; length 70−200 mm), *E. charcoti* (total *n* = 48; length 40−130 mm) and *C. georgiana* (total *n* = 48; length 10−46 mm). Numbers used varied based on the tolerances and total number of individuals available for each species. Within each species, animals were randomly distributed between salinity treatments. Each experimental trial was conducted in a single 30 or 50 l tank fitted with a nanoprotein skimmer (REEF-Skim Nano 100AC, TMC) and bio-filter (ZB−150, Ziss) with an attached airline. Distilled water was mixed with filtered natural seawater until experimental salinity levels were achieved. Tank water was maintained at −0.3 ± 0.5°C. Salinity was measured using a conductivity probe (CDC40101, Hach). For each treatment, oxygen consumption, activity, coelomic fluid osmolality (at 6 and 24 h) and mortality were assessed. After 24 h in experimental salinities, animals were immediately moved to an ambient (34.5‰) seawater tank for observation over a minimum 5 day recovery period.

### (c) Mortality

Following the 24 h exposure and minimum of 5 days recovery in ambient salinity (34.5‰), animals were examined for signs of life (observed movement of tube feet/spines/tentacles) and tested for an activity response (ability to right in *S. neumayeri*, *O. validus* and *O. victoriae*, peristaltic movement in the three holothurians) over several hours using video recording. Animals that showed no signs of life were considered dead.

### (d) Oxygen consumption

Oxygen consumption was measured using closed chamber techniques as described in Barrett *et al*. [27]. Briefly, individual animals (*n* = 5–8 per treatment) were immersed directly into experimental salinity tanks and placed within open respirometry chambers (ranging from 80 to 500 ml depending on the size of the animal). Animals were left for 1 h prior to measurements. Once chambers were sealed, oxygen concentration was measured using a Fibox-4 oxygen sensor (PreSens, Germany). After 4–6 h, a second measurement was taken. Three empty chambers were measured to account for background oxygen changes. All experiments were carried out at mid-morning to minimize any circadian rhythm effects. Individual wet mass (± 0.01 g) and volume (± 0.01 g) were recorded for each animal. To obtain ash-free dry mass (AFDM; ± 0.01 g), all animals were euthanized by freezing at −20°C and dried in a convection oven at 60°C to constant mass (± 0.01 g). Dried animals were transferred to a muffle furnace and heated to 475°C for 6 h. AFDM was obtained by subtraction and used to estimate the mass of respiring organic tissue.

### (e) Activity

The righting response in echinoids, asteroids and ophiuroids is a widely used metric for assessing their response to environmental stress [17,53]. The time taken for animals (*n* = 5 per salinity treatment) to fully right themselves after 6 h in experimental salinities was recorded and converted to an activity coefficient (AC = 1000/righting time in seconds) (after [53]). Maximum time allocated was 60 min for *S. neumayeri* and *O. validus*, which equals a minimum AC value of 0.28 s^−1^ (1000/3600 s), while for *O. victoriae* (which can right in a few seconds) 15 min were allocated, equalling a minimum AC value of 1.11 s^−1^ (1000/900 s).

Two of the three holothurian species (*H. steineni* and *C. georgiana*) in the current study exhibit external pentaradiate symmetry, displaying five equally spaced ambulacra with tube feet [15]. This made righting difficult to assess, necessitating a novel activity assessment. In all three species, after a minor disturbance (e.g. emersion and immersion), movement commenced via peristaltic locomotory waves, as recorded in other holothurians [54]. Each peristaltic wave begins at the animal’s posterior and moves to the anterior. A pre-experimental trial was used to assess whether the number of peristaltic waves within 1 h was a repeatable behaviour following emersion and immersion of *H. steineni* and *C. georgiana*. Behavioural repeatability measures variation among individuals versus variation among measurements within individuals (as per Wolak *et al*. [55]). Six animals of each species were isolated in floating trays, and each animal was alternately emersed and immersed. The number of peristaltic waves observed within 60 min was recorded. Each trial was repeated in succession (three in *C. georgiana* and four in *H. steineni*). A *ca* 20 min gap was given between trials, comparable to previous studies on echinoderm righting repeatability [56,57]. The intraclass correlation coefficient (ICC) or *R* (repeatability) values were calculated as:


$$ICC\;or\;R=\frac{S_{A}^{2}}{S_{A}^{2}\;+\;S_{W}^{2}}$$

where $S_{A}^{2}$ is the variance among groups and $S_{W}^{2}$ is the variance within groups. Data were obtained using the R package ICC [55]. The threshold for a repeatable behaviour is commonly taken to be ICC ≥ 0.37 [56–58]. *Heterocucumis steineni* produced ICC = 0.6 (95% CI = 0.21−0.92), with the individual number of waves ranging from 2 to 6 (mean ± s.e.m. = 3.5 ± 0.2). *Echinopsolus charcoti* produced ICC = 0.87 (95% CI = 0.60−0.99), with the individual number of waves ranging from 1 to 5 (mean ± s.e.m. = 3.1 ± 0.3). Upon establishing that this was a repeatable behaviour in *H. steineni and C. georgiana,* the following protocol was utilized in all three holothurian species. After 6 h in experimental salinities (*H. steineni* and *E. charcoti*: *n* = 8 per treatment; *C. georgiana*: *n* = 6 per treatment), each animal was individually removed from the tank and held in the air for *ca* 5 s before reimmersing in the same salinity, and the number of peristaltic waves observed within 60 min was recorded. A photograph was taken every 30 s for detailed assessment. If a wave began at the end of 60 min, it was included as a whole wave to ensure count data were recorded as integers for later analysis.

### (f) Coelomic fluid osmolality

Coelomic fluid samples were taken by non-lethal extraction to assess osmolality at 6 and 24 h after immersion in each experimental salinity, utilizing a different set of animals at each timepoint (except in *C. georgiana* at 9‰ after 24 h, where samples were not collected). Numbers of animal replicates at each salinity were *n* = 3 at 6 h and *n* = 7 or 8 at 24 h for *S. neumayeri*, *n* = 3−5 at 6 h and *n* = 5 or 6 at 24 h for *O. validus*, and *n* = 3 at 6 and 24 h for *H. steineni*, *E. charcoti* and *C. georgiana* (except 9‰ for *C. georgiana* at 6 h, *n* = 2). A syringe with a 21-gauge needle was inserted into the coelomic cavity and *ca* 0.5 ml of coelomic fluid extracted (as per Barrett *et al*. [27]). Triplicate tank water samples were collected at each sample point and a mean seawater osmolality obtained for use in one-way *t*‐test comparisons with coelomic fluid. The osmolality of the coelomic fluid and seawater was measured on a Vapro 5600 vapour pressure osmometer (ELITech Group).

### (g) Statistics

One-way analysis of variance (ANOVA) tests (including Welch’s ANOVA for data with unequal variances) were performed to compare oxygen consumption, osmolality data and activity responses (except for the holothurians) between salinity treatments. Assumptions of normality and equal variance were tested using the Shapiro–Wilk test and Levene’s test, respectively. When assumptions of normality were violated, data were log transformed. When violations of normality persisted, non-parametric Kruskal–Wallis tests were implemented. Significant ANOVA/Kruskal–Wallis were further examined using *post hoc* tests to identify variations among treatments. Tukey tests were used for normally distributed data, Dunn’s test for non-parametric data and Games–Howell tests for data with unequal variance. For assessing the number of peristaltic waves per hour in the holothurians, salinity was treated as a continuous variable and a Poisson model was fitted to assess significance, with a *χ*-squared test to assess goodness of fit. If high overdispersion was observed, a quasi-Poisson model was attempted. To assess significance between salinity treatments, two sample Poisson tests were carried out between all salinity treatment pairings with Bonferroni-adjusted *p* values. The comparison between coelomic fluid values and the mean osmolality of tank water was conducted using one-sample *t*-tests. Second-order regressions were fitted to all 6 and 24 h osmolality data as these consistently produced lower Akaike information criteria (AIC) values and higher *r* ^2^ values when compared with a linear model, in addition to the clear non-linear arrangement of the 6 h data. However, additional linear models were fitted to the 24 h data to assess the degree of osmoconforming. LC_50_ values were calculated by fitting a linear model to the mortality data across salinities from the point prior to where mortality first occurred to where mortality reached 100%. Statistical tests were considered significant if *p* < 0.05. All analysis were carried out in R (v. 4.2.3).

## 3. Results

### (a) Mortality

After 24 h in experimental salinities, mortalities were recorded at ≤19‰ in *O. victoriae,* at ≤14‰ in *S. neumayeri, O. validus* and *H. steineni*, and at 9‰ for *E. charcoti* and *C. georgiana* (electronic supplementary material, table S1). For *O. victoriae*, *S. neumayeri* and *H. steineni*, mortalities were observed directly after the 24 h period with no animals showing any signs of recovering when placed back in ambient salinity (34.5‰). For *O. validus*, some individuals survived for up to 7 days in ambient salinity, including a single animal at 9‰. For *E. charcoti and C. georgiana*, all animals survived for at least 7 days after the 9‰ trial before dying, with one individual of *E. charcoti* surviving for 14 days before dying.

The calculated LC_50_ value is 19.9‰ for *O. victoriae,* 16.5‰ for *S. neumayeri*, 13.4‰ for *H. steineni* and *O. validus* and 11.5‰ for *E. charcoti* and *C. georgiana* (electronic supplementary material, table S1).

### (b) Oxygen consumption

**Figure 1.**
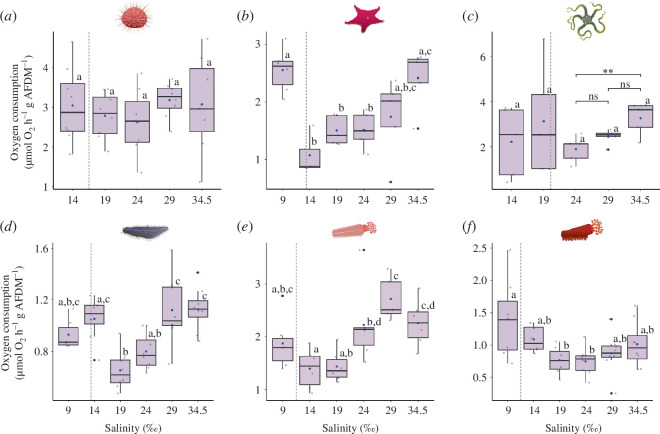
Oxygen consumption measured over the first 5–7 h after transfer from ambient salinity (34.5‰) in six Antarctic echinoderms: (*a*) *Sterechinus neumayeri*, (*b*) *Odontaster validus*, (*c*) *Ophionotus victoriae*, (*d*) *Heterocucumis steineni*, (*e) Cucumaria georgiana*, (*f*) *Echinopsolus charcoti.* Results are shown for one-way ANOVA and *post hoc* Tukey test, with different letters indicating significant differences (*p* < 0.05; *n* = 7 or 8 (*a*), *n* = 5 (*b, c*), *n* = 8 (*d, f*), *n* = 5 or 6 (*e*) biological replicates for each treatment) between treatments. Bars in (*c*) show results between 24 and 34.5‰ only (ns, not significant; **p < 0.01). Box plots show medians (black horizontal line), upper and lower quartiles, and maximum and minimum (whiskers). Biological replicates are represented by grey dots, means by blue dots and outliers by black dots. Grey dashed line represents the calculated LC_50_ value.

Salinity had a significant impact on oxygen consumption in *O. validus* (one-way ANOVA: *F*_5,25_ = 9.1, *p* < 0.0001), *C. georgiana* (one-way ANOVA: *F*_5,29_ = 9.2, *p* < 0.0001), *H. steineni* (one-way ANOVA: *F*_5,40_ = 6.2, *p* < 0.001) and *E. charcoti* (one-way ANOVA: *F*_5,40_ = 3.7, *p* = 0.008) (figure 1; electronic supplementary material, table S2). For *O. victoriae,* there was no significant impact on oxygen consumption when all five treatments were included; however, on removal of the lowest two salinity treatments (14 and 19‰), there was a significant decline in oxygen consumption between 34.5 and 24‰ (one-way ANOVA: *F*_2,12_ = 7.3, *p* = 0.008; Tukey 34.5‰ versus 24‰, *p* = 0.007). Salinity had no significant impact on oxygen consumption in *S. neumayeri*. *Post hoc* testing demonstrated that oxygen consumption was significantly lower compared with animals in controls at salinities 24, 19 and 14‰ in *O. validus*, at 24 and 19‰ in *H. steineni*, and at 14 and 19‰ in *C. georgiana.* At very low salinities, oxygen consumption was significantly higher at 9‰ compared with 14, 19 and 24‰ in *O. validus*, at 9‰ compared with 19‰, and at 14‰ compared with 19 and 24‰ in *E. charcoti,* and at 14‰ compared with 19‰ in *H. steineni* (electronic supplementary material, table S2). Across all species, there were no significant differences in oxygen consumption between the lowest salinity treatment (14‰ in *S. neumayeri* and *O. victoriae*; 9‰ for all other species) and the control (electronic supplementary material, table S2).

### (c) Activity

**Figure 2.**
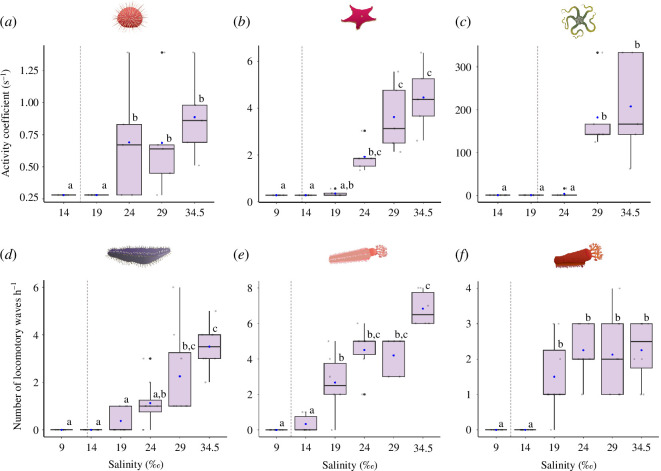
Activity rates 6 h after transfer from ambient salinity (34.5‰) into low salinity treatments in six Antarctic echinoderms; (*a*) *Sterechinus neumayeri*, (*b*) *Odontaster validus*, (*c*) *Ophionotus victoriae*, (*d*) *Heterocucumis steineni*, (*e) Cucumaria georgiana*, (*f*) *Echinopsolus charcoti.* (*a–c*) Righting ability was converted to an activity coefficient, calculated as AC = 1000/righting time in s; a smaller AC value indicates a longer righting time. (*d–f*) Number of peristaltic locomotory waves recorded over 1 h. Results are shown for Kruskal–Wallis and *post hoc* Dunn’s test (a–c), and two-sample Poisson test (*d*–*f*) with different letters indicating significant differences (*p* < 0.05; *n* = 5 (*a*–*c*), *n* = 6 (*e*), *n* = 8 (*d, f*) biological replicates for each treatment). Box plots show medians (black horizontal line), upper and lower quartiles, and maximum and minimum (whiskers). Biological replicates are represented by grey dots, means by blue dots and outliers by black dots. Grey dashed line represents the calculated LC_50_ value.

Salinity had a significant impact on activity values across all species (*S. neumayeri*: Kruskal–Wallis *$\chi_{4}^{2}$* = 14.58, *p* < 0.01; *O. validus*: Kruskal–Wallis $\chi_{5}^{2}$ = 26.29, *p* < 0.0001; *O. victoriae*: Kruskal–Wallis *$\chi_{4}^{2}$* = 21.27, *p* < 0.001; *C. georgiana*, *E. charcoti* and *H. steineni*: all Poisson *p* < 0.0001) (figure 2, electronic supplementary material, table S2).

Mean AC values were significantly different from the control salinity (34.5‰) at ≤19‰ in *S. neumayeri* and *O. validus*, and ≤24‰ in *O. victoriae*. For the holothurians, the number of locomotory waves recorded in 1 h was significantly different from the control salinity (34.5‰) at ≤24‰ in *H. steineni*, ≤19‰ in *C. georgiana* and ≤14‰ in *E. charcoti* (figure 2; electronic supplementary material, table S2).

### (d) Coelomic fluid osmolality

Coelomic fluid osmolality was significantly affected by salinity in each echinoderm species after 6 h (all *p* < 0.01) and after 24 h immersion (all *p* < 0.0001) (see electronic supplementary material, table S2).

**Figure 3.**
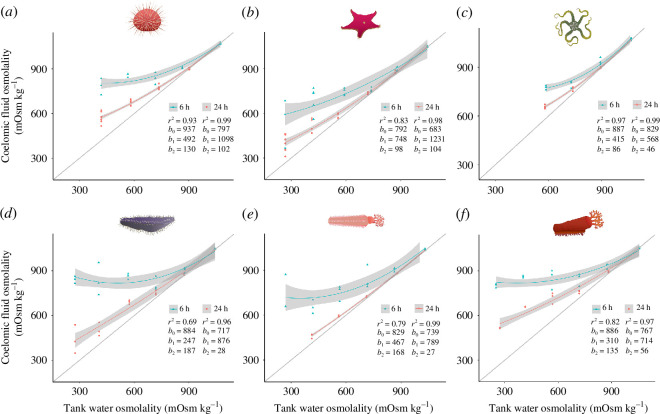
Coelomic fluid osmolality of Antarctic echinoderms at 6 and 24 h after transfer from ambient salinity (34.5‰) versus that of the corresponding tank water; (*a*) *Sterechinus neumayeri*, (*b*) *Odontaster validus*, (*c*) *Ophionotus victoriae*, (*d*) *Heterocucumis steineni*, (*e*) *Cucumaria georgiana*, (*f*) *Echinopsolus charcoti*. Number of animal replicates at each experimental salinity treatment were *n* = 3 at 6 h and *n* = 7 or 8 at 24 h for *S. neumayeri*, *n* = 3−5 at 6 h and *n* = 5 or 6 at 24 h for *O. validus*, *n* = 3 at 6 and 24 h for *H. steineni*, *E. charcoti* and *C. georgiana* (except 9‰ for *C. georgiana* at 6 h, *n* = 2). Mean (± s.e.m.) tank water osmolality (mOsm kg^−1^) in each salinity experiment was: 34.5‰, 1051 ± 6; 29‰, 876 ± 3; 24‰, 721 ± 2; 19‰, 576 ± 2; 14‰, 414 ± 2; and 9‰, 269 ± 3. A second-order polynomial regression line (blue line) and the s.e.m. (shaded grey area) were fitted to the data. The iso-osmotic line is represented by a grey line. Regression coefficients and *r* ^2^ values are included to the right of the isosmotic line.

Coelomic fluid osmolality was either iso- or hyperosmotic to the tank water under all salinities in each species (figure 3). Mean osmolality gradients (difference between coelomic fluid and external tank seawater) after 6 h in salinity treatments were significant at salinities ≤29‰ in *S. neumayeri*, *E. charcoti* and *C. georgiana* (except treatments 24 and 9‰ in *C. georgiana,* which were not significant), at ≤24‰ in *O. victoriae*, at ≤19‰ for *H. steineni* and 14‰ only for *O. validus* (figure 3; see electronic supplementary material, tables S2 and S3 for mean osmolality gradients). The most osmoconforming species at 6 h was *O. validus,* which did not have significant gradients between the coelomic fluid and tank water except at 14‰ (figure 3; electronic supplementary material, tables S2 and S3). After 24 h, mean osmolality gradients were significant at ≤24‰ in *S. neumayeri*, *O. validus*, *O. victoriae* and *E. charcoti,* at ≤19‰ in *C. georgiana* and at 19‰ only in *H. steineni* (figure 3; electronic supplementary material, tables S2 and S3).

After 24 h at 14‰ (the lowest measured treatment where species survived), *E. charcoti* had the largest mean osmolality gradient compared with other species (244 ± 1 mOsm kg^−1^), which was significantly different from all other species, excluding *O. victoriae* where data were absent (Tukey test: all *E. charcoti* comparisons *p* < 0.05) (electronic supplementary material, table S2).

Linear regressions of the 24 h data were used to assess the most osmoconforming species (all had *r* ^2^ values ≥0.96) (electronic supplementary material, figure S1). *Cucumaria georgiana* was the most osmoconforming species, with the closest slope value to 1 (*b*_1_ = 0.93) and the lowest intercept (*b*_0_ = 73) of all species. The most osmoconforming species to the least were: *C. georgiana* > *O. victoriae* > *O. validus* > *H. steineni* > *S. neumayeri *> *E. charcoti*.

## 4. Discussion

Short-term low salinity tolerance varied dramatically between the six Antarctic echinoderm species examined. The brittle star, *O. victoriae*, showed the least tolerance to low salinity, with 80% mortality at 19‰ after 24 h exposure. In contrast, two holothurian species, *E. charcoti* and *C. georgiana*, demonstrated 100% survival for at least a week after 24 h in 9‰ before dying. At lower salinities, all species exhibited reduced activity and the development of a temporary osmotic gradient between coelomic fluid and external seawater. The typical metabolic pattern in all species exhibited a depression in oxygen consumption across tolerated low salinities; however, at extremely low salinities, oxygen consumption increased to levels similar to those at ambient salinities, and this increase corresponded to critical salinity tolerance limits.

### (a) Mortality and LC_50_

Evaluating the tolerance limit to low salinity has been a prominent feature in echinoderm salinity studies [16,17]. This assessment provides a reference point of where the salinity gradient becomes lethal, leading to the organism's demise, while also aiming to determine whether such limits align with the degree of freshening that species may encounter in their natural habitats [17,18].

In previous studies on asteroids, the record for the lowest tolerated salinity was observed in *P. regularis*, which survived at 0‰ for up to 4 days [21]. However, this is considered exceptional, and the majority of lower tolerance limits range from 14 to 27‰ [16]. Therefore, *O. validus*, which tolerated ≥14‰, demonstrates a comparatively high tolerance of hypoosmotic stress relative to other species of asteroids.

In a global review of echinoids, Russell [16] showed lower tolerance limits are in the range of 14−27‰. For example, *Echinus esculentus* survived for 25 days at 21‰ and had an estimated 24 h LC_50_ of 18.5‰ [27]. The majority of studies have used longer durations to assess LC_50_, for example Stickle *et al*. [59] observed a 28 day LC_50_ of 13 and 21‰ for *Strongylocentrotus droebachiensis* and *Strongylocentrotus pallidus,* respectively. The results reported here for adult *S. neumayeri* (24 h LC_50_ = 16.5‰) suggest that it has moderate resilience to acute hypoosmotic stress relative to other echinoids, and contrasts with its extremely stenohaline embryonic form, where 99% mortality was recorded at 30‰ [46].

The Antarctic holothurians demonstrated the greatest resilience to low salinity out of the four echinoderm classes. However, there was notable interspecies variation. Mortality in *H. steineni* (from ≤14‰) was observed immediately after 24 h exposure, marked by evident epithelial rupture and pigment loss. Interestingly, *E. charcoti* and *C. georgiana* initially appeared to recover from 9‰, demonstrating full peristaltic movements for at least 7 days prior to mortality. Examples of high tolerance to low salinity have been observed in other holothurians, for example *Eupentacta quinquesemita* survived for 30 days at *ca* 12.5‰ [29], while other studies report tolerance ranging from 14 to 26‰ [16,17]. In this respect, Antarctic holothurians appear exceptionally tolerant of low salinity relative to other holothurians.

In contrast, the ophiuroid *O. victoriae* was the least euryhaline species examined, with a calculated 24 h LC_50_ *ca* 20‰. Interestingly, ophiuroids are considered to have the greatest tolerance to low salinity of any echinoderm class [17], with examples of species endemic to brackish water environments (e.g. *Ophiophragmus filograneus* [60]). Indeed, laboratory studies demonstrate that *O. filograneus* is able to survive six weeks of continuous exposure to 10‰, with those at 14‰ demonstrating full acclimation [20]. Whether *O. victoriae* is capable of acclimating to lower salinity is currently untested, but the present study suggests that it deviates from previous predictions about ophiuroid tolerance [17].

### (b) Oxygen consumption

Changes in oxygen consumption in response to external stressors are indicative of the need to either utilize more energy to respond to moderate stress (in the case of an increase) or conserve energy in response to extreme stress (in the case of a decrease) [13]. In the current study, four out of the six species demonstrated significant reductions in oxygen consumption between 34.5 and 19‰, while for *O. victoriae* reductions were between 34.5 and 24‰. This suggests that when salinity decreases from ambient levels to the lower end of the animal’s tolerance range, it is characterized by metabolic depression—a reduction in energy expenditure and activity in response to extreme stress [13]. This is likely caused by the large osmotic gradient drawing water into tissue and causing swelling. By reducing physiological activity and diverting energy expenditure away from digestion, growth, reproduction and other processes, energy resources can be solely allocated to homeostatic maintenance, thus maximizing survival chances until favourable osmotic conditions return [13]. Reductions in oxygen consumption in response to acute hypoosmotic immersion have been observed in other echinoderms, for example in echinoids, including *S. purpuratus* [61], *S. droebachiensis* [29] and *E. esculentus*, [27] and a holothurian, *E. quinquesemita* [29].

Common to most species was the noticeable sharp increase in metabolism to levels similar to controls when animals were exposed to salinities past the lower end of their tolerance range. This was most evident in *E. charcoti*, *O. validus* and *H. steineni*, where there were significant differences in oxygen consumption between the lowest salinity treatments and those preceding them. Furthermore, across all species mean oxygen consumption at the lowest salinity was similar to that at ambient salinity. This indicates that a crucial threshold had been crossed, prompting an uptake in metabolic activity to maximize survival. Mechanisms driving the rise in oxygen consumption at these very low salinities are likely distinct from those at ambient salinity. In ambient conditions, these will comprise the whole organism routine energy demands (e.g. maintenance, growth, activity, reproduction, storage etc.), which are expected to remain suppressed under high osmotic stress [13]. Instead, other processes appear to be upregulated, resulting in the net increase in oxygen consumption. These could include processes regulating cell volume adjustment (e.g. the reduction of intracellular free amino acids [62]), the implementation of cellular stress kinase pathways leading to apoptosis [14], and even stress responses to mechanical cell lysis due to extreme swelling. Indeed, the LC_50_ value is closely associated with the salinity treatment where oxygen consumption spikes, suggesting that salinity reduction has passed a critical tipping point.

In the case of *S. neumayeri*, the lack of significant change in metabolic rate was surprising. The absence of a metabolic depression (as observed in all other species examined) may indicate that *S. neumayeri* is well adjusted to low salinity immersion and can rapidly reallocate resources to maintain homeostasis. Indeed, *S. neumayeri* is considered to have one of the lowest recorded metabolic rates of any echinoid [50], which may provide a buffering effect for the initial shock response.

### (c) Activity

Activity rates have been widely used in echinoderm studies to assess functional wellbeing [17]. Righting time is the most commonly used metric to assess responses to environmental stress, especially in asteroids, echinoids and ophiuroids [17], and occasionally holothurians [56]. However, as righting ability was difficult to measure in two of the three holothurian species (*H. steineni* and *C. georgiana*), a peristaltic wave counting metric was employed to assess activity rates under reduced salinity. This novel metric, which is highly repeatable, may be of use in future studies in assessing activity rates in holothurian species without a central orientation, while also applicable to those that do.

Common to all species was a significant decline in activity in relation to reduced salinity. Previous studies have observed similar findings in relation to low salinity, for example in the echinoids *S. droebachiensis* [63] and *E. esculentus* [27], in the ophiuroid *Ophiothrix angulata* [64] and the asteroids *Leptasterias hexactis* [65] and *Pisaster ochraceus* [23]. In the holothurian *Holothuria scabra*, reductions in salinity from 35 to 20‰ induced burrowing behaviour, while at 15‰ there was a reduction in the ability to burrow [66]. The effects of rapid influxes of osmotically drawn water into tissue are likely to cause cellular swelling and appear to disrupt neuro-muscular coordination in echinoderms [63]. Furthermore, the reduction in metabolic activity implies the animals are in a state of energy preservation, prioritizing homeostatic mechanisms over activity levels. Interestingly, even when metabolic rate is elevated at salinity levels near the animal’s critical tolerance threshold, there is no apparent increase in activity. Increased activity at these very low salinities may have been predicted as a mechanism for the animal to seek higher salinities (e.g. [44]). Instead, the lack of activity, despite a high metabolic rate, suggests there is a direct physiological and behavioural cost at very low salinity. Similar increases in oxygen consumption and reductions in activity at very low salinities have been observed in copepods, attributed to the energetic costs associated with reducing the intracellular osmolyte pool to maintain osmotic balance [67]. The current study suggests similar processes may be present in echinoderms at very low salinity.

### (d) Coelomic fluid osmolality

At ambient salinity, all species had coelomic fluid osmolality that was either isosmotic or slightly hyperosmotic to seawater, consistent with findings from other echinoderms [30–32]. After 6 h in reduced salinity, coelomic fluid was hyperosmotic in all species, with wide intraspecies variation. Osmotic gradients that persist for several hours after exposure to reduced salinities have been widely noted in previous echinoderm studies [22,27,32]. It has been suggested that echinoderms may have control over the permeability of epithelial membranes, enabling the establishment of ionic and osmotic gradients [32]. This is hypothesized to protect internal tissue from varying salinity, aligning with cyclical tidal fluctuations [22,32]. However, the formation of osmotic gradients under low salinity is not necessarily associated with an intertidal lifestyle. For example, the intertidal echinoid *Echinometra lucunter*, which is able to tolerate unstable salinity conditions, demonstrated a lack of an osmotic gradient over 35–25‰ [31]. All echinoderm species in the current study are largely subtidal, suggesting that the ability to maintain high osmotic gradients could be the result of prior adaptation or may even serve a different purpose.

After transfer to lower salinities, it is commonly considered to take between 12 and 24 h for coelomic fluids to equilibrate with the external medium [30]. This was observed at 29‰ only in all species after 24 h in the current study. Below this salinity level, large gradients were still present across the majority of species after 24 h. It appears 24 h was insufficient for full osmotic rebalancing at lower salinities, similar to results observed in *E. esculentus* at low salinities [27]. Notably, the least osmoconforming species, *E. charcoti*, maintained coelomic fluid osmolality equivalent to a salinity of *ca* 17‰ (516 ± 2 mOsm kg^−1^) after 24 h in 9‰. Further research is necessary to establish whether the mechanism that enables the maintenance of this remarkable gradient is mediated through control over the permeability of epithelial membranes, ion specific transport [32] or an unknown mechanism. Furthermore, the ability to maintain a large osmotic gradient may not be the sole reason for high tolerance to low salinity, as both the least and most osmoconforming echinoderms (*C. georgiana* and *E. charcoti*, respectively) demonstrated the same LC_50_ of 11.5‰ after 24 h. Santos *et al*. [32] showed that urchins with the least capacity to maintain a high osmotic gradient (therefore the most osmoconforming) exhibited the greatest capacity to regulate tissue volume (i.e. the subtidal echinoid *Arbacia lixula*). The ability to resist tissue swelling through cell volume regulation under hypoosmotic shock may be an important mechanism untested in this study. Indeed, *O. validus* has previously been observed to have control over body volume on exposure to reduced salinity [45]. This may explain its high tolerance to acute low salinity while also being a strong osmoconformer, similar to other echinoderms (e.g. *A. lixula* [32]).

Interestingly, at 6 h exposure to reduced salinities, coelomic fluid osmolality was at similar levels at ≤24‰ in all species. For example, the mean coelomic fluid osmolality of *E. charcoti* after 6 h in 24‰ was 845 ± 25 mOsm kg^−1^, while after 6 h in 9‰, it was 805 ± 6 mOsm kg^−1^ (Games–Howell, 24‰ versus 9‰: *q* = 2.3, *p* = 0.65). This suggests that there is a temporal limitation on osmotic adjustments, regardless of the size of the osmotic imbalance, perhaps owing to constraints on diffusion. However, oxygen consumption (which was also measured over the first 6–7 h) increased to comparable levels with the control at the lowest salinities, departing from the observed metabolic depression seen at salinities in between. This suggests that although internal tissues are buffered from the full osmotic gradient, there must be a physiological cost elsewhere, perhaps from exposed external epithelial tissue such as tube feet. With a lack of a buffer, epithelial cells are exposed to the full osmotic gradient (e.g. the coelomic fluid of an echinoderm acclimated to 34.5‰ is ~1050 mOsm kg^−1^, while 9‰ seawater has an osmolality of ~270 mOsm kg^−1^, therefore the osmotic gradient is ~780 mOsm kg^−1^). Therefore, various processes, from volume regulation to cell damage responses, as previously described, may account for the increased metabolic rate at very low salinities. Further work is needed to assess the impact of very low salinities on external echinoderm epithelia and the extent to which they can volume regulate.

### (e) Ecological perspective and conclusions

This study is the first to our knowledge to assess the impact of acute low salinity on four of the major classes of Antarctic echinoderms. They were collected from the subtidal (10–20 m) in Ryder Bay, Adelaide Island, on the Antarctic Peninsula. Here, subtidal salinity at 15 m has remained relatively stable over the past ~20 years at 33.4 ± 0.4‰ (mean ± s.d.) ranging from 32.2 to 34.1‰ [49]. However, closer to the shoreline, the intertidal zone has more dramatic variations in salinity, where run-off from melting ice can expose benthic organisms to salinities down to 12‰ [52], and lower if species become trapped in a hyposaline lens or meltwater stream (e.g. [24,25]). Of the six echinoderms, *S. neumayeri* and *O. validus* are occasionally found in the intertidal [51,52].

The results observed in this study demonstrate that Antarctic echinoderms are not uniformly osmoconformers, and that wide species variations exist in their salinity tolerances. Interestingly, the pattern of tolerance did not correlate with habitat as originally hypothesized. Factors such as phylogenetic or intrinsic biological traits over habitat are likely influencing species tolerance. *Heterocucumis steineni* and *O. validus* exhibit a relatively broad tolerance to changes in salinity, whereas *E. charcoti* and *C. georgiana* display exceptional short-term tolerance to low salinity for echinoderms. These species are expected to demonstrate significant resilience to rapid freshening within the Antarctic environment, both presently and in the future. Indeed, they are unlikely to experience levels of very low salinity if populations remain in the deeper subtidal. It may be predicted that they will simply migrate to deeper, higher salinity waters if freshening persists, potentially impacting ecosystem dynamics negatively [47]. Although predominately subtidal, *S. neumayeri* is known to occasionally inhabit the low intertidal, where salinity is already known to drop to as low as 12‰. This is well below the calculated LC_50_ for this species, perhaps accounting for its preference for deeper water. Furthermore, *S. neumayeri* embryos are highly sensitive to reductions in salinity [46], likely restricting larval recruitment to deeper, higher salinity environments. The most stenohaline echinoderm examined, *O. victoriae*, would certainly be vulnerable to the impact of acute freshening. Its vulnerability to climate change is compounded by its inability to acclimate to warming at +2°C, which is currently only <0.5°C above summer maximum temperatures at Rothera [68]. When multiple environmental stressors are applied in tandem (e.g. warming and low salinity), the effect on organisms can be additive or even synergistic [69,70]. Multiple stressor studies on Antarctic echinoderms have so far not included freshening, which, for a phylum clearly vulnerable to low salinity, should be a focus for future research. Furthermore, attention should be paid to Antarctic crinoids and their ability to tolerate hyposalinity. Worldwide, crinoids are the least studied of all echinoderm classes in relation to salinity tolerance, and have not been found living in low salinity environments [16], suggesting they may be vulnerable to future Antarctic freshening.

The aim of the current experiment was to examine the hypoosmotic shock response to low salinity through acute direct transfer. However, alongside the current approach, incorporating a more gradual, step-wise dilution may better represent the ecologically realistic scenario of acute low salinity in the Antarctic. This method, which could be described as a diurnal acute response, combined with longer-term acclimation experiments, would help decouple the different mechanisms underlying the capacity for low salinity tolerance in Antarctic echinoderms.

Climate change poses concern regarding the increased frequency of freshening events in nearshore coastal ecosystems [4]. Fjordic regions, such as the Western Antarctic Peninsula, are particularly vulnerable to future freshening events due to the presence of marine terminating glaciers [71]. These marine areas are considered to be highly biodiverse [9], and the Antarctic region as a whole is considered a biodiversity hotspot for echinoderms [34]. However, there is fear that future warming will increase freshwater input into Antarctic fjords, diminishing species richness and diversity, as has been observed in Arctic fjords [9]. Ultimately, for Antarctic echinoderms to prosper in a reduced salinity environment over the long term, they will need to demonstrate the ability to acclimatize. Capacity for acclimatization in Antarctic marine species is considered paramount under a climate change context [33,72]. Many are slow growing and have long generation times, meaning that survival will depend on their flexibility to adjust through acclimatization and ensure populations survive long enough for genetic adaptation to take place [33]. Priority now should be focused on the capability for Antarctic echinoderms to acclimatize to low salinity that reflects future climate change projections.

In summary, these findings highlight the species-specific response of Antarctic echinoderms to short-term low salinity, providing valuable insights into their potential resilience to climate-change-induced freshening.

## Data Availability

The datasets supporting this article have been uploaded as part of the electronic supplementary material, table S2 [73].
